# Lateral Curvature of the Spine and Pott’s Disease

**Published:** 1899-02

**Authors:** A. M. Phelps

**Affiliations:** New York City


### Lateral Curvature of the Spine and Pott's Disease.

BY A. M. PHELPS, M. D., NEW YORK CITY.

In presenting this subject to you I wish to give some practical points which, although not new, must be remembered in order to treat these cases intelligently. It has been but a few years since the treatment of these affections was a bugbear to the general practitioner, who sent every case to the specialist. Now, however, with a better understanding of what they are and their remedy, he attends them himself.

Regarding Pott's disease, any stated paper would take at least a week to read. I shall lay before you some material and a few facts, which will cover the subject, well. So many conditions are described as Pott's disease that it is first fitting to say that the true one is tubercular, unquestionably; those not tubercular are not Pott's. The disease was described by Pott as tubercular disease of the ends of the bone or cartilages. Following typhoid fever, there may be acute pain from absorption of the results of inflammatory changes in Peyer's patches, but it is septic. Then mycosis may afford opportunities for mistakes, or a child may suffer an injury, an abscess may result with development of septic symptoms, but this trouble is osteo-myelitis, not Pott's disease.

*Etiology.*—It is a true infection of the bacillus tuberculosis into a focus of previous inflammatory action; that is inoculates tissues not embryonic is impossible. As the area of inflammation extends inoculation takes place, with a destruction of bone, formerly termed caries, but this term had better be dropped. The disease may attack the intervertebral cartilages. Why is it that of two children receiving an injury one will develop Pott's disease and the other not? Because the former is stumous. Stuma is a condition of the protoplasm making up the ultimate cell; it is a state in which one cell succumbs to germ life and the other resists it. It is born with the child, and is seen typically in the slums of any large city, being imported to this country by the people who live in the walled cities of Europe. It will take America a thousand years to grow children free from this condition.

Here is a preserved specimen of the spine taken from a patient with Pott's disease, in which the vertebra is destroyed and its neighbors are consolidated, showing the projection of the spinal processes posteriorly. It is typical. Here is one of rheumatoid arthritis, which might have been taken for Pott's disease. This illustration shows destruction of the cord from projection of bone into the canal. And here is a case of extreme kyphos, but without pressure on the cord.

*Diagnosis.*—Lateral curviture differs from Pott's disease in that it is never produced by inflammation or disease of the spinal column except rickets. Positions in utero produce it, as in short leg. Frequently it is tried to diagnose lateral curvature when Pott's disease is present. It is a symptom of the latter. A diagnosis of this kind would result disastrously, because the treatment of the two differs. It must also be diagnosed from pseudo-hypertrophic paralysis.

Before deformity begins is the correct time to make the diagnosis. (After deformity has occurred it is easy.) In the beginning there is difficult breathing (often treated for asthma or worms). If the child is lifted it will cry. It has "bellyache," and it holds its hands flexed to its side. There never was disease of a joint in which motion was not limited from muscular spasm. If the spine is flexed it is not Pott's disease; if it is rigid you may be absolutely certain it is. This boy (exhibiting a negro youth) has a flexible spine, although lateral curvature is present. If a baby has Pott's disease, and you raise it by its head, it will come up stiff; if not it will roll up like a ball.

There is always pain (usually anterior) in Pott's disease, but not in lateral curvature. If high up it is in the chest; lower, in the stomach; still lower, in the abdomen. After the case has gone on there occurs muscular atrophy and then deformity, but no swelling. On one side the bodies of the vertebræ are absorbed, but on the opposite side they are normal.

There is not a single straight spine in the world; if so a man would break his head every time he jumped six feet. Every lateral curvature to be cured must have a compensating curve, so as to allow the vertical through the center of gravity to fall between the feet. In some patients, e. g., those with rickets, the curvature is due to pressure. Ossification is sometimes due to central-nerve lesion. Other curvatures may be caused by injury.

*Treatment* must be based on rational principles. I would treat lateral curvature with gymnastics and a support to remove pressure. Pott's disease is to have the same treatment as a broken leg, i. e., fixation, to give nature a chance to repair.

*Lateral Curvature.*—Some say that every brace produces atrophy; others that bracing is all that is required to remove pressure and prevent absorption. Bracing, properly done, does not produce atrophy. A very good plan before beginning treatment is to determine the extent of bone changes. Have the patient to bend forward; then the application of a straight line along the back will show the extent of deviation. Find the size of the vertebræ, and then brace. The diameter of the column is usually two inches. If deviation is one-half of this, a mechanical appliance is absolutely necessary to obtain stable equilibrium, producing thus one curve to balance the other.

A child under three years cannot be braced, for the pelvis is too small as compared with the thorax, and the retaining strap will slip. Put on a Sayre's cuirasse or a plaster of Paris portable bed. The latter is also of benefit in Pott's and hip disease. I got the idea from observing an Indian squaw carrying her baby.

Regarding spinal bracing, where the band around the pelvis is narrow and small there is tilting, I believe that suspension and then fixation is necessary. The Hessing corset was invented in 1764, and forcible replacement in 1768, and then abandoned, and we will have to do likewise. Sayre was the first man in this country to make a suitable apparatus for Pott's disease and lateral curvature—the plaster of Paris corset. Notwithstanding that it is heavy, cumbersome and wears out, it is the best of all braces. He marked a new era in the treatment of these diseases when he suspended the patient and thus removed the pressure. Afterwards it was sought to use other materials, and then came leather and rawhide, which proved valueless.

I went to Odessa to learn to make the wood corset, and was pleased with it, but as soon as perspiration occurred its shape changed and the patient became shorter. Then I invented the apparatus which I here exhibit, viz.: the aluminum corset. Its life is from fifteen to twenty years. It was first made in lateral halves, but, proving cumbersome, the duplex hinge was added, and now it can be put on and laced as the ordinary corset. In lateral curvature, with proper gymnastics, it will cure.

The new operation of forcible replacement was used by Hippocrates 500 B. C., forgotten, revived in the time of Ambroise Pare (fifteenth century), again forgotten, and finally revived recently. Any authority commenting upon it says the results are too good to be credited. I am very sure that old cases with anchylosis, great deformity or abscess, should not be touched. In beginning cases, pushing and then treatment as described before may avail, but the vertebræ must be wired. Even then in two or three years they will be found bent.—*N. Y. Acad. Med. Trans.*

